# Mutations in retrotransposon *AtCOPIA4* compromises resistance to *Hyaloperonospora parasitica* in *Arabidopsis thaliana*

**DOI:** 10.1590/S1415-47572009005000099

**Published:** 2010-03-01

**Authors:** Yi-Hong Wang, James T. Warren

**Affiliations:** 1 School of Science, Behrend College, Penn State University, Erie, PAUSA; 2 Department of Renewable Resources, University of Louisiana, Lafayette, LAUSA

**Keywords:** *Arabidopsis thaliana*, retrotransposon, downy mildew resistance, knockout

## Abstract

Retrotransposons (RTEs) are a principal component of most eukaryotic genomes, representing 50%-80% of some grass genomes. RTE sequences have been shown to be preferentially present in disease resistance gene clusters in plants. *Arabidopsis thaliana* has over 1,600 annotated RTE sequences and 56 of these appear to be expressed because of the exact expressed sequence tag (EST) matches and the presence of intact open reading frames. Of the 22 represented in the Affymetrix ATH1 array, *AtCOPIA4* was found to be expressed at a higher level than all other RTEs across different developmental stages. Since *AtCOPIA4* is located in the *RPP5* gene cluster and is adjacent to *RPP4* which confers resistance to the downy mildew oomycete *Hyaloperonospora parasitica* isolate EMWA1, we evaluated *AtCOPIA4* mutants for resistance to this pathogen. T-DNA insertional and antisense knockout of *AtCOPIA4* was found to reduce the resistance of wild type plants by 2-4 folds. Our results suggest that retrotransposon can be exapted to participate in plant defense response.

Retrotransposons (RTEs) are a principal component of most eukaryotic genomes, representing more than 40% of the human genome (Kazazian, 2004; Lander *et al.*, 2001) and 50%-80% of some grass genomes (Feschotte *et al.*, 2002; SanMiguel and Bennetzen, 1998). Even in the compact genome of *Arabidopsis thaliana*, they account for 5.5% of the sequenced genome (Kazazian, 2004). Cellular functions of RTEs have been reported. They seem to play a role in the proliferation of cancer cells (Oricchio *et al.* 2007), globally interfere with the regulatory network of transcription factors p53 (Wang *et al.*, 2007) and PSF (Song *et al.*, 2005), and interact with the dynein light-chain which is a known component of the dynein microtubule motor (Havecker *et al.*, 2005). In mammals, RTEs are more likely to be found in rapidly evolving gene clusters, such as those involved in defense and response to external signals, than in mRNAs of highly conserved genes involved in development, transcription, replication, cell structure and metabolism (Medstrand *et al.*, 2005; van de Lagemaat *et al.*, 2003). In plants, the pattern is similar. For example, *Tos17* retrotransposon is preferably inserted into disease/defense-related and signal transduction (kinase) genes in the rice genome (Miyao *et al.*, 2003). Furthermore, RTEs have been identified in disease resistance gene clusters in lettuces (Meyers *et al.*, 1998; Michelmore and Meyers, 1998), rice (Song *et al.*, 1995), barley (Marcel *et al.*, 2007), the common bean (Vallejos *et al.*, 2006), poplar (Lescot *et al.*, 2004), and Arabidopsis (van der Biezen *et al.*, 2002; Yi and Richards, 2007). Various RTEs have been shown to be induced by plant pathogens or elicitors in rice (Chen *et al.*, 2007; Vergne *et al.*, 2008), by *Fusarium oxysporum* in chickpea (Nimbalkara *et al.*, 2006), and by fungal elicitors in tobacco (Pouteau *et al.*, 1994; Melayah *et al.*, 2001). In addition, RTE *Tnt1A* inserted in a tobacco resistance gene cluster has been shown to drive partial transcription of the neighboring disease resistance gene *TNLL1* (Hernández-Pinzón *et al.*, 2009).

RTE coding sequences are also known to form chimeric transcripts (Kashkush *et al.*, 2003; Peaston *et al.*, 2004) with non-RTE mRNA sequences and chimeric transcripts displaying a different expression pattern from that of the original transcripts (Peaston *et al.*, 2004). Chimeric resistance and retrotransposon genes may function in disease resistance. For example, *L10* is a Toll/Interleukin1 receptor-nucleotide binding site-leucine-rich repeat [TIR-NBS-LRR] class of resistance gene (Lawrence *et al.*, 1995) and a chimera of the *L10* TIR domain fused with a partial tobacco retrotransposon sequence at the 3' end has been reported. Expression of this chimera caused the same stunted phenotype produced by over-expressing full-length *L6*, and increased transcript abundance of a constitutive defense protein PR-1 (Frost *et al.*, 2004). Similarly, *Xa21D* truncated at the 3' end with only the extracellular LRR domain by the retrotransposon *Retrofit* confers partial resistance to the bacterial pathogen *Xanthomonas oryzae* pv *oryzae* (Wang *et al.*, 1998).

Here we show that knocking out the Arabidopsis retrotransposon *AtCOPIA4* (*At4g16870*; Yi and Richards 2007) reduces resistance to the downy mildew pathogen *Hyaloperonospora parasitica* isolate EMWA1. *AtCOPIA4* is represented in a single copy of the Arabidopsis genome based on BLAST search, and is located next to *RPP4*, separated only by its long terminal repeat (LTR; Figure 1). *AtCOPIA4* protein contains the conserved domains of gag-integrase-reverse transcriptase. *In silico* EST analysis identified a chimeric cDNA consisting of the first exon of *RPP4* which encodes the complete TIR domain upstream from the partial sequence of *AtCOPIA4* (Figure 1), similar in configuration to the resistance gene domains truncated downstream by RTEs described above. Pathogenicity assays demonstrated that T-DNA insertional and antisense RNAi mutants were 2 to 4 times as likely to be infected by *H. parasitica* isolate EMWA1 to which Arabidopsis *RPP4* (At4g16860), a TIR-NB-LRR class of disease resistance gene, confers host resistance (van der Biezen *et al.*, 2002).

Potential *AtCOPIA4* T-DNA insertional mutant SALK_005767 in the Col-0 background (Alonso *et al.*, 2003) was obtained from the Arabidopsis Biological Resources Center at Ohio State University. To identify a homozygous insertion plant, two PCR reactions with primers LP+RP and LB+RP were set up using Ex Taq from Takarabio USA (Madison, WI). PCR was run with initial denaturing at 94 °C for 2 min and 35 cycles of 94 °C/30 s, 58 °C/30 s and 72 °C/2 min., followed by 72 °C for 5 min. A single PCR product from LB and RP primers was amplified and sequenced to determine the exact T-DNA insertion site in homozygous plants. Position of T-DNA insertion was thus determined and indicated in Figure 1. One heterozygous and one homozygous plant were identified and used in the study. Primers used for plant identification were:

LB: GCGTGGACCGCTTGCTGCAACT

LP: CTACTGATGTATTGTTGCCAGAGG

RP: ATCTCCGTAATAGAGGGAGTGTTG

Antisense RNAi plants were generated through the transformation of antisense sequence of *AtCOPIA4* (see Figure 1), by using primers A1 (AACTAAAGACGAGCT CTATGAATG) and A2 (TCTAGATTAATGAAACAAT CCGAACAAG) which contain restriction sites for *Sac*I and *Xba*I, respectively. The amplified PCR product was first cloned into a TA cloning vector pGEM T Easy (Promega, WI), and then into the binary vector pBI121 digested with *Sac*I and *Xba*I. Arabidopsis Col-0 transformation followed a floral-dip protocol as described (Clough and Bent, 1998). T_2_ transgenic plants were used in the pathogenicity assay.

For RT-PCR analysis, total RNA was isolated from two week-old *Hyaloperonospora parasitica* EMWA1 infected seedlings using TRIzol (Invitrogen, CA), and then treated with DNase I (Ambion, TX) according to manufacturer's protocol. RT-PCR was performed using the Verso 1-Step RT-PCR kit (Thermo Scientific/Fisher, PA). PCR was run for 15 min at 50 °C, 15 min at 95 °C, followed by 25 cycles of 95 °C/30 s, 58 °C/30 s, 72 °C/2 min, and the final extension of 5 min at 72 °C. All RT-PCR primers were tested for their target specificity using Seqviewer (www.arabidopsis.org). All the primers used showed desired specificity:

P1: GTAGATGTTCGCAAAACGTTCCTC

P2: AATCACCATTTGTTCCCCTTTCTT

P3: TTAAGAGCAAGACCTTGAGATGGC

P4: GAGGACAAACCAGAGGATCAGAAA

P5: TGTTGCTCCAAGGGAGAACTAAAG

P6: ATGAAACAATCCGAACAAGCAAGT

UBQ1: GATCTTTGCCGGAAAACAATTGGAGGATGGT

UBQ2: CGACTTGTCATTAGAAAGAAAGAGATAACAGG

To conduct pathogenicity assay, seeds were planted in soils (Metromix 360, SunGro, Canada) saturated with water and stratified at 4 °C for 48 h. Pathogenicity assays followed those described previously (Holub *et al.*, 1994; Yoshioka *et al.*, 2006). Briefly, 10 to 14 day-old conidiophores of *H. parasitica* isolate EMWA1 (kindly provided by Daniel Klessig) were collected from susceptible live plants of Nd-0 and re-suspended in cold, sterile water. The spores were vortexed for 30 s for release from the sporangia. Spore concentration was adjusted to 10^4^-10^6^ per mL, and 1-2 μL of the spore suspension was dropped onto each cotyledon of 6 to 7 day-old plants (10 to 20 plants for each line in each replicate). The inoculated plants were covered with plastic wrap and incubated at 16 °C with 10 hour-photoperiods. At 10 to 14 days after inoculation, the number of conidiophores on each cotyledon leaf, number of cotyledon leaves with conidiophores and the total number of plants, were recorded using a dissection microscope. The experiment was replicated three times with similar trends. Both resistant (Col-0) and susceptible (Nd-0; Holub *et al.*, 1994) lines were used as pathogenicity assay controls, although only Col-0 data are shown in Figure 2 and Figure 3.

To identify Arabidopsis retrotransposons that had acquired cellular functions, we searched the genome sequences of about 1,600 annotated retrotransposon genes curated in VirtualPlants (virtualplant.bio.nyu.edu; www.virtualplant.org) for matches to ESTs. Among these, 56 had exact matches to EST sequences and intact open reading frames. Twenty two of the genes were represented in the Affymetrix Arabidopsis ATH1 GeneChip and expression of those was searched in over 3,000 GeneChips in the Genevestigator database (Zimmermann *et al.*, 2004). *AtCOPIA4* was selected because it represents a typical retrotransposon which encodes gag, integrase and reverse transcriptase proteins (Feschotte *et al.*, 2002), and it is the most highly expressed retrotransposon throughout the development stages, although generally their expression level is low due to regulation by the host. *AtCOPIA4* transcript level was found to be highest in developing leaves and flowers (Table 1). This expression pattern was also confirmed by the Massively Parallel Signature Sequencing (MPSS) mRNA signature data (Nakano *et al.*, 2006). Genes in this region have been shown to be co-expressed, probably due to local chromatin structural changes (Yi and Richards, 2007; Zhan *et al.*, 2006). Cluster analysis in Genevestigator also revealed that *AtCOPIA4*, *RPP4* and *At4g16880,* which are three adjacent genes on chromosome 4 based on the latest genome annotation release (TIGR/AGI V8) and Yi and Richards (2007), were coexpressed under salt, cold, heat, wound, oxidative, and genotoxic conditions (data not shown). Correlation of *AtCOPIA4* expression is 0.59 with *At4g16880* and 0.55 with *RPP4*, as calculated in the ATTED-II database of Arabidopsis microarray data (Obayashi *et al.*, 2007). This correlation between *AtCOPIA4* and *RPP4* is noticeable in Table 1 as well.

To elucidate the function of *AtCOPIA4*, a homozygous T-DNA insertion mutant was identified from SALK_005767 and antisense RNAi mutants were generated, as described above. Sequencing analysis indicated that T-DNA was inserted 70 bp before the start codon of *AtCOPIA4* and 117 bp after the stop codon of *RPP4* in SALK_005767 (Figure 1) in the LTR. RT-PCR analysis of the mutant seedlings indicates that the *AtCOPIA4* transcript was undetectable in the homozygous T-DNA insertional mutant (I10) but present in the heterozygote (I3; Figure 2), indicating that transcription of *AtCOPIA4* had been knocked out in the T-DNA insertion mutant. Among the two antisense mutants tested (A60, and A80), *AtCOPIA4* transcript levels were undetectable in A60 and significantly reduced in A80 (Figure 2). In the mutants with no or reduced *AtCOPIA4* transcript, the level of the *AtCOPIA4*-*RPP4* chimeric transcript was also either not apparent or was at a reduced level (Figure 2). However, the abundance of *RPP4* transcript was not affected in these lines, when compared to Col-0 and based on RT-PCR analysis, using primers P3 and P4, as shown in Figure 1 (Figure 2).

No noticeable morphological difference was observed between the mutants and Col-0. However, because *AtCOPIA4* is located in the cluster of *RPP5* class of resistance genes (van der Biezen *et al.*, 2002; Yi and Richards, 2007), right next to *RPP4* and *in silico* EST analysis had revealed a chimeric *AtCOPIA4*-*RPP4* mRNA (Figure 1), we sought to evaluate the mutants for resistance to *H. parasitica* isolate EMWA1. Pathogenicity assays showed that on average, homozygous insertional and antisense mutants were 2 to 4 times as likely to be infected by the isolate based on percentage of infected plants, whereas heterozygous insertional mutants were as resistant to the isolate as Col-0 (Figure 3). Notwithstanding, the number of conidiophores was not significantly higher in the mutants examined 10 days after inoculation, when compared to wild type Col-0. Overall, the number of conidiophores ranges from 2 to 5 per cotyledon leaf on average for all lines and replicates and the highest number of conidiophores was 15 found in the mutants.

How *AtCOPIA4* functions in the disease resistance is not clear. *AtCOPIA4* may contribute to resistance to *H. parasitica* isolate EMWA1 either through the chimeric transcript (TIR-RTE) or through other mechanisms, since knockout undermines resistance conferred by *RPP4*. Even so, *RPP4* transcript level was not noticeably different among the five lines tested (Figure 2). It has previously been shown that *L10* TIR-RTE chimeric expression increases *PR-1* transcription (Frost *et al.* 2004) and that a chimeric *Xa21D*-*Retrofit* confers partial resistance to *Xanthomonas oryzae* pv *oryzae* (Wang *et al..* 1998). Retrofit is 41% identical and 57% similar to AtCOPIA4, based on a comparison of the whole protein sequence, thus making *Retrofit* the most homologous RTE from another species to *AtCOPIA4*. The coding region of *Retrofit* contains all the domains as in *AtCOPIA4* and the truncated *Xa21D* encodes the LRR domain (Song *et al..* 1997; Wang *et al..* 1998). Both LRR and TIR domains affect resistance gene specificity in plants (Ellis *et al..* 1999; Luck *et al..* 2000). Apparently, expression of these domains alone could have an impact on disease resistance. While the chimera of *L10* TIR-RTE and *Xa21D*-*Retrofit* are caused by RTE insertion in the DNA sequence, the *RPP4* TIR-*AtCOPIA4* fusion is due to the fact that the two genes are adjacent and chimeras formed at the RNA level (Figure 1). Therefore, it will be of interest to see whether increasing *RPP4*-*AtCOPIA4* chimera expression would boost resistance as conferred by *RPP4* because the level of the chimerical transcript was much lower in the mutants tested (Figure 2). *AtCOPIA4* expression is driven by the 130 bp LTRs flanking the coding region. Future studies should focus on how the chimeric transcript is generated with the *AtCOPIA4* sequence downstream from the *RPP4* TIR domain, in contrast to what has been reported in other cases.

We have shown here that knockout of an RTE compromises plant resistance to the downy mildew pathogen *H. parasitica* EMWA1. RTEs have been shown to play a role in defense response in other eukaryotes as well. In mammals, degraded reverse transcribed RTEs can trigger defense response from the immune system (Stetson *et al.*, 2008). Our evidence suggests that RTEs also function in defense response in plants.

**Figure 1:**
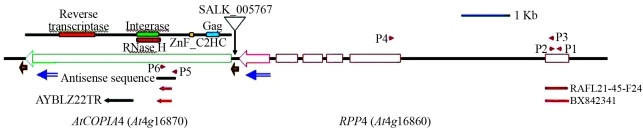
The Arabidopsis genomic region of *AtCOPIA4* (in green) and *RPP4* (in red) based on Yi and Richards (2007) who have sequenced the full-length cDNA of genes in this region. Open boxes represent exons and lines between boxes represent introns in *RPP4*. *AtCOPIA4* conserved domains are indicated above the gene. Location of T-DNA insertion is indicated for SALK_005767. Antisense sequence represented by a black line below was used for an *AtCOPIA4* antisense construct. One cDNA match (AYBLZ22TR) to *AtCOPIA4* is also shown. Chimeric cDNAs are drawn in red broken lines and arrows (RAFL21-45-F24 and BX842341). Two chimeric ESTs were also identified in GenBank: ES444452 and EL142415 (not shown). Affymetrix GeneChip probes for both genes are shown in blue arrows. Brown open arrows below the ends of *AtCOPIA4* are the 130 bp long terminal repeats (LTRs; 9488607-9488478 and 9483894-9483755, respectively).

**Figure 2:**
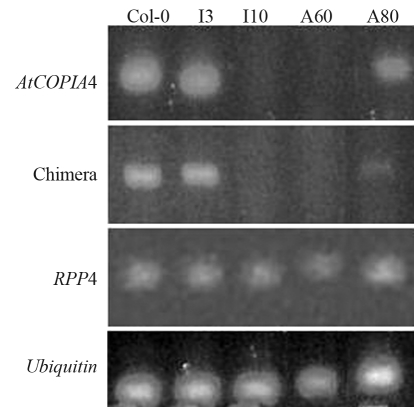
RT-PCR of Arabidopsis T-DNA insertion and antisense mutants in *AtCOPIA4*. Primers used for *AtCOPIA4* are P5 and P6, P1 and P6 for the chimeric transcript as shown in Figure 1 and P3 and P4 for RPP4. Total RNA from seedlings was used. Lines used are: Col-0-Columbia wild type; I3, I10-heterozygous and homozygous T-DNA insertion lines, respectively; A60, and A80 are antisense lines. Chimera indicates *RPP4*- *AtCOPIA4* chimeric mRNA.

**Figure 3:**
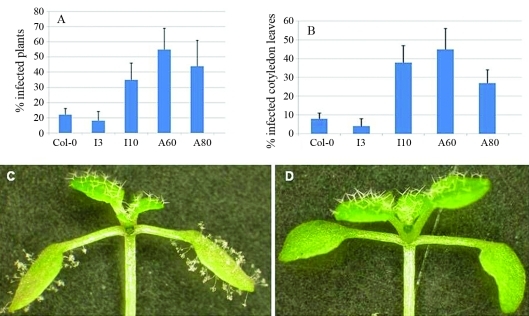
Percentage of infected Arabidopsis wild type and mutant plants 10 days after inoculation of *Hyaloperonospora parasitica* EMWA1. (A) Percent of infected plants. (B) Percent of infected cotyledon leaves. (C) and (D) show an infected I10 and an uninfected wild type plant, respectively. Lines tested are: Col-0-Columbia wild type; I3, I10-heterozygous and homozygous T-DNA insertion lines, respectively; A60, and A80 are antisense lines.

## Figures and Tables

**Table 1:** Average signal intensity of selected retrotransposon genes in different developmental stages in Arabidopsis^*a*^.

	Germinated seed	Seedling	Young rosette	Developed rosette	Bolting	Young flower	Developed flower	Flowers and siliques	Siliques
*AtCOPIA4* (At4g16870)	310	619	646	741	503	681	699	450	460
*RPP4* (At4g16860)	80	432	1,053	978	380	1,291	771	332	536
At3g21020	295	166	154	170	314	216	268	205	280
At2g15510	125	145	134	147	112	193	195	124	201
At2g17490	23	20	17	18	43	19	27	14	20
*ACT2* (At3g18780)	14,828	18,867	15,468	16,580	13,033	14,243	13,732	16,403	5,333
Total arrays^*b*^	169	944	419	173	150	277	619	121	57

^*a*^Only four of the 22 RTEs are presented in the table. The RTEs are randomly selected (except *AtCOPIA4*) to show that *AtCOPIA4* has the highest transcript abundance. *RPP4* is included as a comparison for its expression pattern with that of *AtCOPIA4*. *Actin2* (*ACT2*) is included as a control. ^*b*^Total number of arrays (GeneChips) used to obtain the averaged signal for each stage. Data are gathered from the Genevestigator database (https://www.genevestigator.ethz.ch).
